# Genetic Variants Associated with Thyroid Cancer Risk: Comprehensive Research Synopsis, Meta-Analysis, and Cumulative Epidemiological Evidence

**DOI:** 10.1155/2021/9967599

**Published:** 2021-12-14

**Authors:** Ran Ran, Gang Tu, Hui Li, Hao Wang, Exian Mou, Caiyang Liu

**Affiliations:** ^1^Department of Breast Surgery, Sichuan Cancer Hospital and Institute, Sichuan Cancer Center, School of Medicine, University of Electronic Science and Technology of China, Chengdu 610041, China; ^2^Department of Endocrine Breast Surgery, The First Affiliated Hospital of Chongqing Medical University, Chongqing 400016, China; ^3^Department of Cardiothoracic Surgery, The First People's Hospital of Neijiang, Neijiang 641000, Sichuan, China

## Abstract

**Purpose:**

With the increasing incidence of thyroid cancer (TC), associations between genetic polymorphisms and TC risk have attracted a lot of attention. Considering that the results of associations of genetic variants with TC were usually inconsistent based on publications until now, we attempted to comprehensively evaluate the real evidence of associations between single nucleotide polymorphisms (SNPs) and TC risk.

**Method:**

We performed meta-analyses on 36 SNPs in 23 genes associated with TC susceptibility based on the data from 99 articles and comprehensively valued the epidemiological evidence of significant associations through the Venice criteria and false-positive report probability (FPRP) test. OR and *P* value were also calculated for 19 SNPs in 13 genes based on the insufficient data from 22 articles.

**Results:**

19 SNPs were found significantly associated with TC susceptibility. Of these, strong epidemiological evidence of associations was identified for the following seven SNPs: POU5F1B rs6983267, FOXE1 rs966423, TERT rs2736100, NKX2-1 rs944289, FOXE1 rs1867277, FOXE1 rs2439302, and RET rs1799939, in which moderate associations were found in four SNPs and weak associations were found in eight SNPs. In addition, probable significant associations with TC were found in nine SNPs.

**Conclusion:**

Our study systematically evaluated associations between SNPs and TC risk and offered reference information for further understanding of polymorphisms and TC susceptibility.

## 1. Introduction

Thyroid cancer (TC) is the most common endocrine malignant tumor with the increasing incidence worldwide. Besides radiation exposure, TC is also closely related to family inheritance and genetic variant risk [1]. As early as 2009, Gudmundsson et al. firstly pointed out that variants on 9q22.33 (FOXE1) and 14q13.3 (NK2 homeobox 1 (NKX2-1)) might increase the risk of papillary thyroid cancer and follicular thyroid cancer [2]. BRAF V600E mutation is comparatively common and widely used in the detection of papillary thyroid cancer [3]. However, still most of the genetic variation remains uncharacterized with TC susceptibility.

So far, the research on associations between genetic variation and cancer risk received a lot of attention. Quite a few pooled studies and reviews have expounded the relationship between TC and genetic variation [4–6], but it is difficult to interpret the inconsistent results between the same variants and TC risk. A small sample size may not have sufficient ability to detect the true associations. Meta-analyses can comprehensively conduct secondary research by collecting the effective data from single study, which can increase the statistical power and reliability of the causality [7, 8]. However, there are still inconsistent results in the meta-analyses updated until now, which indicates the existence of false-positive report caused by unnecessary overlap. Moreover, the Venice rating standard, firstly proposed by Ioannidis et al. [9], has been used to systematically grade the cumulative evidence of genetic associations, so as to help understand associations between genetic variants and disease [10, 11]. Herein, we collected data updated until now and performed meta-analyses to comprehensively evaluate the evidence for further understanding of associations between genetic variation and TC risk.

## 2. Materials and Methods

Our study was performed based on the guidelines of the Preferred Reporting Items for Systematic Reviews and Meta-Analyses (PRISMA) Statement and the Human Genome Epidemiology Network for the systematic review of genetic association studies [12, 13].

We searched publications about genetic variation and TC risk on PubMed, MEDLINE, Web of Science, and CNKI before December 31, 2020, using the keywords as follows: (“thyroid”), (“cancer” or “carcinoma”), (“genetic” or “single nucleotide polymorphism (SNP)” or “SNP” or “polymorphism” or “genotype” or “variation” or “variant” or “mutation” or “susceptibility”), (“association” or “associate”), using “and” collect each keyword as well. A total of 3887 records were searched, as well as 157 records from relevant reference publications. As a result, 99 relevant publications with available data were included in our study. The articles included in our study must meet the following inclusion criteria: (i) the object of study must be thyroid cancer, (ii) studying associations between genetic variants and etiology of TC using human-related case-control or cohort or cross-sectional study, and (iii) offering sufficient data to perform meta-analyses. Repetitive and unrelated articles were excluded by browsing titles and abstracts. The articles were excluded (i) if the interests were not concentrated on variants with TC risk, (ii) if there is lack of necessary data, and (iii) if the articles were just letters to editors.

### 2.1. Data Extraction

Data extraction was carried out by two people independently and exchanged to check with each other after extraction. Any inconsistent was duplicately checked and discussed to reach an agreement with the corresponding author. For the variants reported in articles, we extracted data as follows: PMID of articles, first author, year of publication, country or region, ethnicity, name of variants, polymorphisms, study design, genotyping method, case and control, Hardy–Weinberg equilibrium (HWE) status, genotype counts, and minor allelic frequency (MAF). According to the results of previous meta-analyses, we divided the major ethnic groups into 3 group categories: Caucasians, Asians, and African Americans. The overall population was defined as two or more populations as above. As for the name of SNP, which often has many different naming methods, we selected the most common and well-known name of the SNP as the representative by querying on NCBI.

### 2.2. Statistical Analysis

For each SNP, we sorted out allelic, dominant, and recessive models according to the included ethnicities. Then, meta-analyses based on models and ethnicities were performed using STATA, version 12 (Stata, College Station, TX, USA) only if two or more studies were included. Crude ORs with the corresponding 95% CIs were used to assess the strength of the association between SNPs and TC risk. The *I*^2^ test was performed to quantitatively assess possible heterogeneity in the combined studies as follows: *I*^2^ ≤ 25 indicated no or mild heterogeneity, 25% < *I*^2^ < 50% indicated moderate heterogeneity, and *I*^2^ ≥ 50% indicated large heterogeneity [14]. Sensitivity analysis was performed by removing the first published study from the total or studies deviated from the HWE in the controls and reanalyzing the remainder. In addition, publication bias was assessed by Egger's test and *P* > 0.05 indicated no publication bias existed [15, 16].

### 2.3. Evaluation of Epidemiological Evidence

We evaluated the evidence of significant associations between SNPs and TC by the Venice guideline first based on three criteria as follows: amount of evidence, replication of association, and protection from bias [17, 18]. The amount of evidence was related to the number of alleles or genotypes and graded as A (*N* > 1000), B (100 ≤ *N* ≤ 1000), or C (*N* < 100). The replication of association was graded as A (I2 ≤ 25%), B (25% < I2 < 50%), or C (I2 ≥ 50%) based on heterogeneity statistics. The protection from bias was determined by various potential sources of bias, including sensitivity analysis, publication bias, and small study bias, as well as an excess of significant findings. A was graded when there was no demonstrable bias or the bias was unlikely to invalidate the association. B was graded when there was no obvious bias without sufficient information on identifying evidence. C was graded when there was obvious bias or the bias was likely to explain the presence of association. Furthermore, C was graded in any one of the following situations: (1) association lost with exclusion of first study or studies deviated from HWE in sensitive analysis; (2) a low magnitude of the association (0.87 < OR < 1.15) only if the association had been identified by GWAS or several studies with no evidence of publication bias; and (3) evidence of obvious publication bias (*P* value in Egger's test<0.05). In summary, the cumulative epidemiological evidence of significant associations was graded as follows: strong associations (all above three grades were A), weak associations (any grade was C), and moderate associations (all other conditions).

Furthermore, we used a false-positive report probability (FPRP) assay suggested by Wacholder et al. [17] with a prior probability of 0.05 and an FPRP cutoff value of 0.2 to detect the potential false-positive results among significant associations, in order to confirm whether there was a real association between SNPs and TC risk. The evidence of FPRP was graded as strong (FPRP < 0.05), moderate (0.05 ≤ FPRP ≤ 0.2), and weak (FPRP > 0.2), which indicated upgrading of cumulative evidence one level (from moderate to strong or from weak to moderate), maintaining of the original level, and downgrading of cumulative evidence one level (from strong to moderate or from moderate to weak), respectively.

## 3. Results

**Figure 1:**
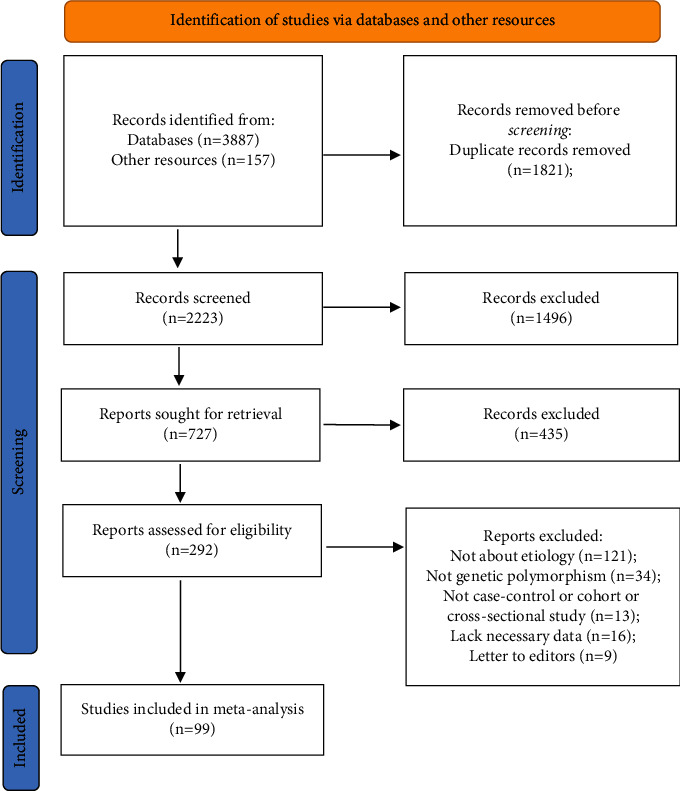
Flow diagram of search strategy and study selection.

As presented in Figure 1, a total of 3887 records were searched, as well as 157 records from relevant reference publications. Of these, 1821 duplicate records were removed, and 1931 irrelevant records were excluded via scanning the title or abstract. Of the 292 publications assessed for eligibility, 121 publications were excluded due to no etiology of TC, 34 publications were excluded due to no genetic polymorphism, 13 publications were excluded due to no case-control or cohort or cross-sectional study, 16 publications were excluded due to lack of necessary data, and 9 publications were excluded for letter to editors. At last, a total of 99 eligible publications were included in our study. The data from those publications involving 36 SNPs in 23 genes were used to perform meta-analyses and value the cumulative epidemiological evidence with the Venice criteria and FPRP test. Additionally, 22 publications including 19 SNPs in 13 genes with insufficient data were also used to calculate OR and *P* value.

In the result of meta-analyses, 19 SNPs were significantly associated with TC risk as follows: POU5F1B rs6983267, miR-146a rs2910164, FOXE1 rs71369530, FOXE1 rs907580, NKX2-1 rs944289, FOXE1 rs965513, FOXE1 rs966423, FOXE1 rs1443434, FOXE1 rs1867277, FOXE1 rs2439302, FOXE1 rs30215269, MTHFR rs1801133, RET rs1800858, RET rs1799939, RET rs1800862, RET rs1800863, TERT rs2736100, XRCC3 rs1799794, and XRCC3 rs861539 (Table 1). 17 SNPs had no obvious association with TC as follows: ATM rs189037, ATM rs664677, ATM rs1801516, CYP1A1m1 rs4646903, CYP1A1m2, GSTM1 null/present, GSTP1, GSTT1 null/present, NAT rs10419839, P53 rs1042522, RET rs2565206, RET rs1800861, XRCC1 rs25487, XRCC1 rs25489, XRCC1 rs1799782, XRCC2 rs3218536, and XRCC3 rs1799796 (Supplementary Table 1). Of the significantly associated SNPs, RET rs1800858 was found inversely associated with TC risk (OR = 0.898 under allelic model and 0.867 under dominant model). All other significant associations of SNPs could increase the risk of TC.

Furthermore, in the result of subgroup analysis for 19 SNPs based on ethnicity, 11 SNPs were significantly associated with TC risk as follows: 3POU5F1B rs6983267, NKX2-1 rs944289, FOXE1 rs965513, DIRC3 rs966423, FOXE1 rs966423, FOXE1 rs2439302, MTHFR rs1801133, RET rs1800858, RET rs1799939, XRCC1 rs1799782, and XRCC3 rs861539 (Table 2), and 8 SNPs were not (miR-146a rs2910164, P53 rs1042522, XRCC1 rs25487, XRCC1 rs25489, XRCC3 rs1799794, XRCC3 rs1799796, RET rs1800861, and RET rs1800863) (Supplementary Table 2). In the result of merely calculating OR and *P* value, probable significant associations with TC were found in 9 SNPs (CYP1A2F rs762551, FTO rs1477196, FTO rs8047395, FTO rs11642841, FTO rs17817288, IL-18 rs360717, miR-608 rs4919510, TSHR rs1991517, and XRCC3 rs56377012) (Table 3).

Sensitivity analysis was performed for all significantly associated SNPs and significant SNPs in subgroup analysis by removing the first published study from the total publications or studies deviated from the HWE in the controls. As a result of removing the first published study, RET rs1800858 was no longer significantly associated with TC under all models, neither was RET 1800862 under the allelic model. In addition, only XRCC3 rs1799794 lost the significant association with TC when removing studies deviating from the HWE. Meanwhile, publication bias was assessed by Egger's test. Obvious publication bias was shown in FOXE1 rs965513 under recessive model for the overall population, RET rs1799939 under recessive model for the Asian population, RET rs1800862 under dominant model for the Caucasian population, XRCC1 rs1799782 under recessive model for the Asian population, and XRCC3 rs861539 under recessive model for the overall population and the Caucasian population.

Next, we assessed the cumulative epidemiological evidence of significant associations through the Venice criteria. Of all the 19 SNPs significantly associated with TC, 3 SNPs were found strongly associated with TC risk (POU5F1B rs6983267, FOXE1 rs966423, and TERT rs2736100), 6 SNPs were found moderately associated with TC risk (NKX2-1 rs944289, FOXE1 rs1867277, FOXE1 rs2439302, MTHFR rs1801133, RET rs1799939, and RET rs1800863), and 10 SNPs were found weakly associated with TC (miR-146a rs2910164, FOXE1 rs71369530, FOXE1 rs907580, FOXE1 rs965513, FOXE1 rs1443434, FOXE1 rs30215269, RET rs1800858, RET rs1800862, XRCC3 rs1799794, and XRCC3 rs861539). As for the subgroup analysis of ethnicity, 4 SNPs were assessed as a strong association with TC (POU5F1B rs6983267, NKX2-1 rs944289, DIRC3 rs966423, and FOXE1 rs966423), 4 SNPs were assessed as a moderate association with TC (MTHFR rs1801133, RET rs1799939, XRCC3 rs861539, and FOXE1 rs965513), and 3 SNPs were assessed as a weak association with TC (FOXE1 rs2439302, RET rs1800858, and XRCC1 rs1799782).

Furtherly, we valued the cumulative epidemiological evidence of associations according to the FPRP value calculated at a prior probability of 0.05 and used the statistical power to detect an odds ratio of 1.5. As a result, 7 SNPs (POU5F1B rs6983267, FOXE1 rs966423, TERT rs2736100, NKX2-1 rs944289, FOXE1 rs1867277, FOXE1 rs2439302, and RET rs1799939) were graded as strong cumulative epidemiological evidence of association with TC risk and 4 SNPs (NKX2-1 rs944289, FOXE1 rs1867277, FOXE1 rs2439302, and RET rs1799939) therein were upgraded from moderate to strong as the FPRP value. 4 SNPs were graded as a moderate association with TC (FOXE1 rs71369530, FOXE1 rs965513, MTHFR rs1801133, and XRCC3 rs861539). Of these, the cumulative epidemiological evidence of 3 SNPs (FOXE1 rs71369530, FOXE1 rs965513, and XRCC3 rs861539) was upgraded from weak to moderate and 1 SNP (MTHFR rs1801133) was maintained as a moderate association. 8 SNPs were graded as weak associations with TC risk (miR-146a rs2910164, FOXE1 rs907580, FOXE1 rs1443434, FOXE1 rs30215269, RET rs1800858, RET rs1800862, XRCC3 rs1799794, and RET rs1800863). Only 1 SNP (RET rs1800863) was downgraded from moderate to weak, and all others were still maintained as weak associations with TC risk.

In addition, in the subgroup analysis, 7 SNPs were graded as strong associations with TC after calculating FPRP value (POU5F1B rs6983267, NKX2-1 rs944289, DIRC3 rs966423, FOXE1 rs966423, MTHFR rs1801133, XRCC3 rs861539, and FOXE1 rs965513), in which the cumulative epidemiological evidence of MTHFR rs1801133, XRCC3 rs861539, and FOXE1 rs965513 was upgraded from moderate to strong. 2 SNPs were graded as a moderate association with TC (FOXE1 rs2439302 and RET rs1799939), and the association of FOXE1 rs2439302 was upgraded from weak to moderate. 2 SNPs were still maintained as weak association with TC based on the FPRP value (RET rs1800858 and XRCC1 rs1799782).

## 4. Discussion

In this study, we collected data about associations between polymorphisms and TC from publications, performed meta-analyses, and valued the cumulative epidemiological evidence of associations by the Venice criteria and FPRP test, which extended our understanding of true associations between SNPs and TC etiology.

DIRC3, first identified as a fusion transcript in familial renal carcinoma as early as 2003, was identified to affect thyroid-stimulating hormone levels and promote TC development through decreasing thyroid epithelium differentiation [18, 19]. The SNP rs966423 located in 2q35 of the DIRC3 gene, within a lncRNA, was valued as strong evidence for association with TC risk in our study. The allele C mutation increased TC risk in the overall population and the Caucasian population compared with the wild-type allele T (OR = 1.227 and OR = 1.214, respectively). However, lack of data resulted in ambiguous associations for the Asian population. As susceptibility genetic loci of DIRC3 were also commonly found in GWAS in the Korean population [20], further investigation for SNPs on DIRC3 in the Asian population is necessary.

The TERT gene is a catalytic subunit of telomerase and plays an essential part in cellular immortality by maintaining telomere length at the end of chromosomes, which exhibited low or no expression in normal cells but highly expressed in 85%–90% of tumor cells and stem cells [21–23]. The SNP rs2736100 is located in intron No. 2 of TERT gene and has a genotype-specific impact on TERT expression [24]. In our meta-analyses, 5 studies with a sample size of over 10000 subjects demonstrated its true evidence of strongly increasing TC risk in the Asian population, especially for the Chinese population. GWAS conducted by Julius Gudmundsson et al. confirmed the similar result in populations of European ancestry (rs2736100(C): OR = 1.11; *P* = 7.3 × 10^4) [25].

The SNP rs6983267 is located in chromosome 8q24 and has been identified to be associated with several cancers, such as prostate, ovary, colon, and several other carcinomas [26, 27]. POU5F1B (also known as POU5F1P1) is the nearest gene of rs6983267, which can probably encode a functional protein contributing to carcinogenesis by acting as a weak transcriptional activator [28]. We found strong epidemiological evidence of increasing TC risk among the overall population, especially in the Caucasian population. A higher TC risk among Caucasians than Asians was demonstrated in our study, and it was consistent with the result of the meta-analyses performed by Zhu et al. [26], which may be related to the lower mutation of risk allele G among Asians than Caucasians.

The SNP rs944289 is located in a 249 kb LD region near the gene of NK2 homeobox 1 neighborhood (NKX2-1), which plays a vital role in thyroid morphogenesis regulating via encoding thyroid transcription factor 1 (TTF1) [27]. Previous studies found that it is significantly associated with TC risk in the Japanese and Icelandic populations, but not associated with that in the Belarusian population [27, 29]. Strong evidence of increasing TC risk among three populations was confirmed in our meta-analyses with over 10000 subjects. Previous publication is referred to a probable relationship between rs944289 and female TC susceptibility for a higher prevalence of allele T in female patients of TC [30].

In our study, RET rs1799939 was found significantly increasing with the TC risk by 1.535-fold and had strong epidemiological evidence in the overall population. A change from allele G to allele A of rs1799939 may activate RET via leading to an amino acid change from glycine to serine, which played a vital role in thyroid carcinogenesis [31, 32].

Forkhead factor E1 (FOXE1), also called TTF2 for thyroid transcription factor (2), was firstly isolated from cDNA of mouse and modified the development of the thyroid gland and their expression in thyroid tumors through encoding thyroid-specific transcription factors [33, 34]. For both rs1867277 and rs2439302, strong accumulative epidemiological evidence of increasing TC risk was demonstrated among Caucasians in our study. Previous publications have referred that allele A of rs1867277 was significantly related to TC risk in Poles [35]. As one of the most specific thyroid transcription factors, FOXE1 could identify thyroperoxidase and thyroglobulin, which contributed a lot in tumor transformation [36], but lack of sufficient data resulted in the ambiguous association among the Asian population, which need further accumulation and investigation about other ethnicities.

4 SNPs in our study were demonstrated as a moderate association with TC risk and 8 SNPs as weak associations. For SNPs such as FOXE1 rs965513, MTHFR rs1801133, XRCC3 rs861539, and XRCC3 rs1799794, a different epidemiological evidence for associations was observed in different ethnicities or genetic models. In addition to ethnic heterogeneity, the influence of diverse genetic behaviors and multiple environments should also be considered in further well-designed studies. Due to insufficient data, only OR and *P* value were calculated for 19 SNPs in which 7 SNPs revealed probably increased TC risk, while 2 SNPs might decrease the risk of TC. Further large size studies were expected to identify the actual association for these SNPs.

A total of 17 SNPs showed no association with TC risk in meta-analyses. A similar result was also found in the meta-analysis of Kang et al. that ATM variants might not be important dominants of TC susceptibility [37]. Besides, 5 SNPs had a sample size of more than 6000 subjects with the MAFs ranging from 10% to 30%. Based on the detection level or value setting at 1.15 in the additive model, the meta-analyses can provide about 86% power with a MAF of 10% and improve 97% power with a MAF of 20%. Therefore, no significant results may be presented for these five SNPs in the future TC susceptibility investigation with a similar sample size.

Certain inevitable limitations existed in this study: (i) despite the full trade-off between inclusion and exclusion criteria, some articles may have been missed; (ii) owing to the insufficient of some data, meta-analyses could not be performed for SNPs included in each ethnicity and genetic model; (iii) study was designed only for associations among SNPs and TC susceptibility, but not involved in tumor progression, metastasis, and prognosis of TC; and (iv) factors included in this study were only ethnicity and genetic models, and other factors such as pathological types of TC and radiation exposure should be considered to further assess the association. Despite these limitations, our study provides an updated and comprehensive evaluation of the TC susceptibility and provides a reference for further genetic research.

In conclusion, our study comprehensively assesses the cumulative epidemiological evidence of significant associations among SNPs and TC susceptibility based on the Venice criteria and FPRP test. Seven SNPs were identified as strong evidence of associations with TC risk, as well as four SNPs with moderate evidence. We provided an updated understanding of TC susceptibility and inspired further investigation into gene polymorphism and clinic strategy of TC.

## Figures and Tables

**Table 1:** Statistically significant variants from meta-analysis, false-positive report probabilities (FPRPs), and cumulative epidemiological evidence.

Gene	Variant	Alleles	Ethnicity	MAF†	Studies	Number evaluation			Risk of meta-analysis			PQ	Amount of evidence		Replication	Protection from bias		Reason for bias exemption	Venice criteria grade§	FPRP values at prior probability of 0.05 and OR of 1.5	Cumulative epidemiological evidence^¶^
						Sample size (case/control)	Genetic models	Effect model	OR (95% CI)	*Pvalue*	*I* (%)		N‡minor	Grade		Grade	Pegger				
POU5F1B	rs6983267	G > T	Overall	0.47	10	30673 (7504/23169)	Allelic	F	1.129 (1.086 − 1.174)	≤0.01	28.1	0.19	29188	A	B	C^a^	0.25	No	ABC	≤0.01	Moderate
					9	27428 (7018/20410)	Dominant	F	1.175 (1.107 − 1.247)	≤0.01	0.0	0.48	20223	A	A	A	0.33	No	AAA	≤0.01	**Strong**
					9	27428 (7018/20410)	Recessive	F	1.158 (1.091 − 1.228)	≤0.01	27.6	0.19	6725	A	B	A	0.28	No	ABA	≤0.01	**Strong**
miR-146a	rs2910164	G > C	Overall	0.32	12	16737 (4857/11880)	Dominant	R	1.144 (1.003 − 1.304)	≤0.01	57.0	≤0.01	8639	A	C	C^a^	0.89	No	ACC	0.46	Weak
FOXE1	rs71369530	>14-Ala vs. ≤14-Ala	Caucasian	0.24	4	1271 (576/695)	Allelic	R	1.836 (1.353 − 2.492)	≤0.01	64.5	0.04	757	B	C	A	0.27	No	BCA	0.02	Moderate
FOXE1	rs907580	C > T	Caucasian	0.26	3	6884 (497/6387)	Allelic	R	1.593 (1.184 − 2.145)	≤0.01	67.6	0.05	3733	A	C	A	0.80	No	ACA	0.11	Weak

NKX2-1	rs944289	C > T	Overall	0.55	17	65182 (9467/55715)	Allelic	F	1.304 (1.255 − 1.355)	≤0.01	45.5	0.02	72278	A	B	A	0.40	No	ABA	≤0.01	**Strong**
					10	16277 (5187/11090)	Dominant	F	1.609 (1.465 − 1.767)	≤0.01	26.3	0.20	12706	A	B	A	0.24	No	ABA	≤0.01	**Strong**
					10	16277 (5187/11090)	Recessive	F	1.414 (1.301 − 1.536)	≤0.01	40.5	0.09	5073	A	B	A	0.14	No	ABA	≤0.01	**Strong**

FOXE1	rs965513	G > A	Overall	0.34	18	61943 (8167/53776)	Allelic	R	1.703 (1.575 − 1.842)	≤0.01	66.7	≤0.01	43701	A	C	A	0.13	No	ACA	≤0.01	Moderate
					9	14348 (3680/10668)	Dominant	R	1.694 (1.429 − 2.010)	≤0.01	68.4	≤0.01	7542	A	C	A	0.17	No	ACA	≤0.01	Moderate
					9	14348 (3680/10668)	Recessive	F	1.954 (1.729 − 2.208)	≤0.01	46.4	0.06	1599	A	B	C^d^	0.02	No	ABC	≤0.01	Moderate
DIRC3	rs966423	T > C	Overall	0.50	5	9604 (4953/4651)	Allelic	F	1.227 (1.153 − 1.306)	≤0.01	0.0	0.44	9931	A	A	A	0.13	No	AAA	≤0.01	**Strong**
FOXE1	rs1443434	T > G	Caucasian	0.39	4	9627 (2453/7174)	Allelic	R	1.392 (1.084 − 1.787)	≤0.01	78.7	≤0.01	7800	A	C	A	0.54	No	ACA	0.20	Weak

FOXE1	rs1867277	G > A	Caucasian	0.39	12	21820 (5654/16166)	Allelic	F	1.503 (1.426 − 1.583)	≤0.01	43.7	0.05	18169	A	B	A	0.47	No	ABA	≤0.01	**Strong**
					6	10702 (2958/7744)	Dominant	R	1.702 (1.352 − 2.143)	≤0.01	65.4	≤0.01	6892	A	C	A	0.21	No	ACA	≤0.01	Moderate
					6	10702 (2958/7744)	Recessive	F	1.703 (1.498 − 1.937)	≤0.01	44.4	0.11	1809	A	B	A	0.20	No	ABA	≤0.01	**Strong**
FOXE1	rs2439302	C > G	Overall	0.35	4	9265 (3146/6119)	Allelic	F	1.325 (1.240 − 1.415)	≤0.01	25.6	0.26	7409	A	B	A	0.42	No	ABA	≤0.01	**Strong**
FOXE1	rs30215269	T > C	Caucasian	0.39	3	6997 (684/6313)	Allelic	R	1.634 (1.254 − 2.127)	≤0.01	62.0	0.07	5555	A	C	A	0.99	No	ACA	0.97	Weak

MTHFR C677T	rs1801133	C > T	Overall	0.30	8	6267 (2902/3365)	Allelic	R	1.418 (1.114 − 1.806)	≤0.01	71.4	≤0.01	4276	A	C	A	0.39	No	ACA	0.12	Weak
					9	7454 (3447/4007)	Dominant	R	1.383 (1.081 − 1.769)	≤0.01	69.0	≤0.01	4337	A	C	A	0.31	No	ACA	0.20	Weak
					8	6267 (2902/3365)	Recessive	F	1.258 (1.081 − 1.464)	≤0.01	43.1	0.09	843	B	B	A	0.95	No	BBA	0.06	Moderate

RET A45A	rs1800858	G > A	Overall	0.31	8	4620 (1867/2753)	Allelic	F	0.898 (0.818 − 0.987)	0.03	18.9	0.28	2885	A	A	C^ab^	0.24	No	AAC	0.33	Weak
					7	4462 (1809/2653)	Dominant	F	0.867 (0.764 − 0.984)	0.03	11.0	0.35	2358	A	A	C^b^	0.55	No	AAC	0.34	Weak

RET G691S	rs1799939	G > A	Overall	0.21	12	6643 (2853/3790)	Allelic	R	1.352 (1.171 − 1.561)	≤0.01	53.8	≤0.01	2934	A	C	A	0.12	No	ACA	≤0.01	Moderate
					12	6643 (2853/3790)	Dominant	R	1.386 (1.155 − 1.664)	≤0.01	55.6	≤0.01	2475	A	C	A	0.63	No	ACA	≤0.01	Moderate
					12	6643 (2853/3790)	Recessive	R	1.535 (1.224 − 1.924)	≤0.01	0.0	0.90	459	B	A	A	0.29	No	BAA	≤0.01	**Strong**

RET S836S	rs1800862	C > T	Caucasian	0.04	14	6654 (2701/3953)	Allelic	F	1.129 (1.008 − 1.409)	0.04	21.8	0.22	637	B	B	C^ab^	0.74	No	BBC	0.88	Weak
					9	5791 (2250/3541)	Dominant	F	1.283 (1.058 − 1.557)	≤0.01	36.6	0.13	500	B	B	C^d^	≤0.01	No	BBC	0.19	Weak
RET S904S	rs1800863	C > G	Overall	0.19	6	3073 (1178/1895)	Recessive	F	1.578 (1.090 − 2.286)	0.02	0.0	0.75	124	B	A	A	0.48	No	BAA	0.43	Weak

TERT	rs2736100	T > G	Asian	0.39	5	10104 (5052/5052)	Allelic	F	1.430 (1.352 − 1.512)	≤0.01	22.6	0.27	8802	A	B	A	0.49	No	ABA	≤0.01	**Strong**
					5	10104 (5052/5052)	Dominant	F	1.535 (1.411 − 1.668)	≤0.01	15.5	0.32	6768	A	A	A	0.95	No	AAA	≤0.01	**Strong**
					5	10104 (5052/5052)	Recessive	F	1.666 (1.509 − 1.839)	≤0.01	0.0	0.72	2034	A	A	A	0.64	No	AAA	≤0.01	**Strong**

XRCC3 A17893G	rs1799794	A > G	Overall	0.29	4	2477 (1106/1371)	Allelic	R	1.275 (1.008 − 1.613)	0.04	71.2	0.02	1519	A	C	C^c^	0.33	No	ACC	0.47	Weak
					4	2477 (1106/1371)	Dominant	R	1.321 (1.023 − 1.705)	0.03	55.4	0.08	1184	A	C	C^c^	0.73	No	ACC	0.43	Weak
					4	2477 (1106/1371)	Recessive	F	1.383 (1.092 − 1.750)	≤0.01	29.8	0.23	335	B	B	C^c^	0.74	No	BBC	0.15	Weak

XRCC3	rs861539	C > T	Overall	0.24	11	5978 (2413/3565)	Allelic	R	1.363 (1.193 − 1.559)	≤0.01	53.9	0.02	3142	A	C	C^d^	0.03	No	ACC	≤0.01	Moderate
					11	5978 (2413/3565)	Dominant	R	1.357 (1.135 − 1.622)	≤0.01	56.9	≤0.01	2570	A	C	A	0.35	No	ACA	0.62	Weak
					11	5978 (2413/3565)	Recessive	F	1.709 (1.428 − 2.046)	≤0.01	19.6	0.26	572	B	A	C^d^	≤0.01	No	BAC	≤0.01	Moderate

F: meta-analysis was performed under the fixed-effects model. R: meta-analysis was performed under the random-effects model. Overall: two or more ethnicities were reported in the study. †Frequency of minor allele in controls. ‡Number of test allele or genotype. §Venice criteria grades are amount of evidence, replication of the association, and protection from bias. ^¶^Cumulative epidemiological evidence as graded by the combination of results from the Venice criteria and FPRP. ^a^The grade of C is given because the OR value is between 0.87 and 1.15, and the association is not replicated by GWAS or GWAS meta-analysis. ^b^The grade of C is given for no significant association existed by excluding the first published study. ^c^The grade of C is given for no significant association existed by excluding studies deviated from the HWE in the controls. ^d^The grade of C is given for significant publication bias (Pegger ≤0.05)

**Table 2:** Significant variants in subtype analysis from meta-analysis, false-positive report probabilities (FPRPs), and cumulative epidemiological evidence.

Gene	Variant	Alleles	Ethnicity	MAF†	Studies	Number evaluation			Risk of meta-analysis			PQ	Amount of evidence		Replication	Protection from bias		Reason for bias exemption	Venice criteria grade§	FPRP values at prior probability of 0.05 and OR of 1.5	Cumulative epidemiological evidence¶
						Sample size (case/control)	Genetic models	Effect model	OR (95% CI)	*Pvalue*	*I* (%)		N‡minor	Grade		Grade	Pegger				
POU5F1B	rs6983267	G > T	Caucasian	0.50	7	47420 (11450/35970)	Allelic	F	1.139 (1.090 − 1.190)	≤0.01	31.2	0.19	24106	A	B	C^a^	0.32	No	ABC	≤0.01	Moderate
			Caucasian		7	23710 (5725/17985)	Dominant	F	1.202 (1.117 − 1.294)	≤0.01	0.0	0.65	17952	A	A	A	0.29	No	AAA	≤0.01	**Strong**
			Caucasian		7	23710 (5725/17985)	Recessive	F	1.177 (1.097 − 1.261)	≤0.01	45.3	0.09	6154	A	B	A	0.31	No	ABA	≤0.01	**Strong**
			Asian	0.35	3	13926 (3558/10368)	Allelic	F	1.095 (1.009 − 1.189)	0.03	36.3	0.21	5082	A	B	C^a^	0.23	No	ABC	0.37	Weak
			Asian		2	3718 (1293/2425)	Dominant	F	1.123 (1.015 − 1.242)	0.02	38.5	0.20	2271	A	B	C^a^	0.20	No	ABC	0.31	Weak

NKX2-1	rs944289	C > T	Caucasian	0.56	13	59074 (7732/51342)	Allelic	F	1.273 (1.220 − 1.329)	≤0.01	31.1	0.13	66909	A	B	A	0.96	No	ABA	≤0.01	**Strong**
			Caucasian		7	13356 (3957/9399)	Dominant	F	1.468 (1.311 − 1.644)	≤0.01	0.0	0.72	10675	A	A	A	0.45	No	AAA	≤0.01	**Strong**
			Caucasian		7	13356 (3957/9399)	Recessive	F	1.378 (1.255 − 1.514)	≤0.01	47.2	0.08	4399	A	B	A	0.34	No	ABA	≤0.01	**Strong**
			Asian	0.41	4	6108 (1735/4373)	Allelic	F	1.429 (1.315 − 1.553)	≤0.01	50.2	0.11	5369	A	C	A	0.97	No	ACA	≤0.01	Moderate
			Asian		3	2921 (1230/1691)	Dominant	F	1.968 (1.663 − 2.328)	≤0.01	0.0	0.80	2031	A	A	A	0.82	No	AAA	≤0.01	**Strong**
			Asian		3	2921 (1230/1691)	Recessive	F	1.544 (1.296 − 1.840)	≤0.01	19.0	0.29	674	B	A	A	0.25	No	BAA	≤0.01	**Strong**

FOXE1	rs965513	G > A	Caucasian	0.35	16	59202 (7025/52177)	Allelic	R	1.747 (1.613 − 1.891)	≤0.01	64.1	≤0.01	42714	A	C	A	0.54	No	ACA	≤0.01	Moderate
			Caucasian		7	11607 (2538/9069)	Dominant	R	1.765 (1.437 − 2.168)	≤0.01	69.6	≤0.01	6683	A	C	A	0.16	No	ACA	≤0.01	Moderate
			Caucasian		7	11607 (2538/9069)	Recessive	F	2.027 (1.780 − 2.307)	≤0.01	49.8	0.06	1471	A	B	A	0.05	No	ABA	≤0.01	**Strong**

DIRC3	rs966423	T > C	Caucasian	0.42	4	7794 (4108/3686)	Allelic	F	1.214 (1.135 − 1.298)	≤0.01	0.8	0.39	6979	A	A	A	0.12	No	AAA	≤0.01	**Strong**

FOXE1	rs966423	T > C	Caucasian	0.42	4	7794 (4108/3686)	Allelic	F	1.214 (1.135 − 1.298)	≤0.01	0.8	0.39	6979	A	A	A	0.12	No	AAA	≤0.01	**Strong**

FOXE1	rs2439302	C > G	Caucasian	0.48	3	6006 (2611/3395)	Allelic	F	1.326 (1.233 − 1.426)	≤0.01	50.4	0.13	6088	A	C	A	0.05	No	ACA	≤0.01	Moderate

MTHFR C677T	rs1801133	C > T	Caucasian	0.23	5	1728 (514/1214)	Allelic	R	1.434 (1.148 − 1.791)	≤0.01	36.5	0.18	897	B	B	A	0.24	No	BBA	0.04	**Strong**
			Caucasian		5	1728 (514/1214)	Dominant	R	1.423 (1.024 − 1.977)	0.04	50.3	0.09	771	B	C	A	0.43	No	BCA	0.52	Weak
			Caucasian		5	1728 (514/1214)	Recessive	F	2.279 (1.545 − 3.363)	≤0.01	0.0	0.89	126	B	A	A	0.70	No	BAA	0.04	**Strong**

RET A45A	rs1800858	G > A	Caucasian	0.28	6	3808 (1440/2368)	Allelic	F	0.895 (0.804 − 0.997)	0.04	0.0	0.45	2134	A	A	C^ab^	0.69	No	AAC	0.46	Weak
			Caucasian		5	3650 (1382/2268)	Dominant	F	0.857 (0.746 − 0.985)	0.03	19.2	0.29	1782	A	A	C^b^	0.49	No	AAC	0.36	Weak

RET G691S	rs1799939	G > A	Caucasian	0.22	8	4971 (1992/2979)	Allelic	R	1.322 (1.103 − 1.584)	≤0.01	60.0	≤0.01	2371	A	C	A	0.24	No	ACA	0.05	Moderate
			Caucasian		8	4971 (1992/2979)	Dominant	R	1.356 (1.060 − 1.736)	0.02	64.3	≤0.01	1979	A	C	A	0.55	No	ACA	0.27	Weak
			Caucasian		8	4971 (1992/2979)	Recessive	R	1.437 (1.117 − 1.849)	≤0.01	0.0	0.91	392	B	A	A	0.65	No	BAA	0.13	Moderate
			Asian	0.15	4	1672 (861/811)	Allelic	R	1.426 (1.116 − 1.822)	≤0.01	40.5	0.17	563	B	B	A	0.12	No	BBA	0.12	Moderate
			Asian		4	1672 (861/811)	Dominant	R	1.444 (1.098 − 1.899)	≤0.01	34.5	0.21	496	B	B	A	0.25	No	BBA	0.21	Weak
			Asian		4	1672 (861/811)	Recessive	R	2.002 (1.192 − 3.362)	≤0.01	0.0	0.61	67	C	A	C^d^	≤0.01	No	CAC	0.55	Weak

XRCC1	rs1799782	C > T	Asian	0.24	5	3388 (1404/1984)	Recessive	R	1.777 (1.245 − 2.537)	≤0.01	53.7	0.07	321	B	C	C^d^	0.93	No	BCC	0.14	Weak

XRCC3	rs861539	C > T	Caucasian	0.31	6	2611 (1029/1582)	Allelic	R	1.252 (1.060 − 1.478)	≤0.01	37.8	0.15	1641	A	B	A	0.12	No	ABA	0.13	Moderate
			Caucasian		6	2611 (1029/1582)	Recessive	F	1.427 (1.114 − 1.826)	≤0.01	0.0	0.42	321	B	A	C^d^	0.02	No	BAC	0.12	Weak
			Asian	0.19	5	3367 (1384/1983)	Allelic	R	1.507 (1.256 − 1.808)	≤0.01	51.8	0.08	1501	A	C	A	0.78	No	ACA	≤0.01	Moderate
			Asian		5	3367 (1384/1983)	Dominant	R	1.485 (1.178 − 1.871)	≤0.01	54.3	0.68	1250	A	C	A	0.31	No	ACA	0.03	Moderate
			Asian		5	3367 (1384/1983)	Recessive	F	2.103 (1.614 − 2.739)	≤0.01	0.0	0.54	251	B	A	A	0.28	No	BAA	≤0.01	**Strong**

F: meta-analysis was performed under the fixed-effects model. R: meta-analysis was performed under the random-effects model. †Frequency of minor allele in controls. ‡Number of test allele or genotype. §Venice criteria grades are amount of evidence, replication of the association, and protection from bias. ^¶^Cumulative epidemiological evidence as graded by the combination of results from the Venice criteria and FPRP. ^a^The grade of C is given because the OR value is between 0.87 and 1.15, and the association is not replicated by GWAS or GWAS meta-analysis. ^b^The grade of C is given for no significant association existed by excluding the first published study. ^d^The grade of C is given for significant publication bias (Pegger ≤0.05)

**Table 3:** OR and P value for probable significant variants with insufficient data.

Gene	Variant	Alleles	Ethnicity	MAF†	Studies	Number evaluation			Risk of meta-analysis			PQ
						Sample size (case/control)	Genetic models	Effect model	OR (95%CI)	*P* value	*I* (%)	
CYP1A2F	rs762551	A > C	Caucasian	0.44	2	688 (170/518)	Recessive	F	1.921 (1.163 − 3.174)	≤0.01	0.0	0.55
FTO	rs1477196	G > A	Overall	0.32	2	2188 (1044/1144)	Allelic	F	1.176 (1.037 − 1.334)	≤0.01	43.7	0.18
FTO	rs8047395	A > G	Overall	0.42	2	2191 (1046/1145)	Allelic	R	1.235 (1.009 − 1.511)	0.04	61.8	0.11
FTO	rs11642841	C > A	Overall	0.18	2	2195 (1046/1149)	Allelic	F	0.784 (0.653 − 0.941)	≤0.01	38.3	0.20
FTO	rs17817288	G > A	Overall	0.44	2	2194 (1045/1149)	Recessive	F	1.410 (1.148 − 1.732)	≤0.01	0.0	0.60
IL-18-127C-T	rs360717	C > T	Overall	0.34	2	721 (130/591)	Allelic	F	1.652 (1.192 − 2.288)	≤0.01	25.4	0.27
miR-608	rs4919510	G > C	Asian	0.42	2	2975 (1193/1782)	Recessive	F	0.813 (0.664 − 0.997)	0.05	0.0	0.40
TSHR	rs1991517	A > C	Overall	0.27	2	1239 (566/673)	Allelic	F	1.250 (1.046 − 1.494)	≤0.01	0.0	0.83
XRCC3	rs56377012	A > G	Asian	0.07	2	1229 (459/770)	Recessive	F	9.421 (4.581 − 19.378)	≤0.01	0.0	0.86

F: meta-analysis was performed under the fixed-effects model. R: meta-analysis was performed under the random-effects model. Overall: two or more ethnicities were reported in the study. †Frequency of minor allele in controls.

## Data Availability

All data generated or analyzed during this study are included in this published article and its supplementary information files.

## Supplementary Materials

Supplementary Materials Supplementary Table 1: insignificant variants from meta-analysis. Supplementary Table 2: insignificant variants in subtype analysis from meta-analysis.
