# Supplementary material for: Genetic Variants Associated with Thyroid Cancer Risk: Comprehensive Research Synopsis, Meta-Analysis, and Cumulative Epidemiological Evidence
Source: J Oncol. 2021 Dec 14;2021:9967599. doi: 10.1155/2021/9967599 (PMC8691982; doi:10.1155/2021/9967599)
Supplement: Supplementary file 1 [file 9967599.f1.docx]

Supplementary table 1 Insignificant variants from meta-analysis

Gene	Variant	Alleles	Ethnicity	MAF[†]	Number studies	Evaluation sample size (case/control)	Genetic Models	effect model	Risk of Meta-Analysis OR(95%CI)	P_{value}	I^2(%)	P_Q
ATM	rs189037	G > A	Overall	0.46	3	2798(1373/1425)	Dominant	F	0.911(0.772-1.073)	0.26	45.1	0.16
ATM	rs664677	C > T	Overall	0.41	4	2110 (978/1132)	Allelic	F	1.044(0.914-1.191)	0.53	0.0	0.77
			Overall		4	2110 (978/1132)	Dominant	F	0.989(0.813-1.202)	0.91	0.0	0.65
			Overall		4	2110 (978/1132)	Recessive	F	1.188(0.924-1.526)	0.18	25.1	0.26
ATM	rs1801516	G > A	Caucasian	0.12	6	5727 (2385/3342)	Allelic	R	0.894(0.680-1.176)	0.42	62.5	0.02
			Caucasian		7	7204 (2977/4227)	Dominant	R	0.894(0.704-1.134)	0.35	60.4	0.02
			Caucasian		6	5727 (2385/3342)	Recessive	R	0.761(0.493-1.174)	0.22	0.0	0.86
CYP1A1m1	rs4646903	T > C	Caucasian	0.18	4	1927 (247/1680)	Allelic	R	1.214(0.787-1.872)	0.38	66.6	0.03
			Caucasian		6	2843 (690/2153)	Dominant	R	0.922(0.622-1.368)	0.69	71.2	≤0.01
			Caucasian		4	1927 (247/1680)	Recessive	R	1.546(0.830-2.877)	0.17	0.0	0.74
CYP1A1m2		A > G	Caucasian	0.26	3	973 (366/607)	Dominant	F	1.061(0.803-1.402)	0.68	0.0	0.42
GSTM1		null vs present	Caucasian	0.51	12	4303 (1417/2886)	Allelic	F	1.032(0.902-1.182)	0.64	34.3	0.12
GSTP1		A > G	Caucasian	0.24	5	1716 (588/1128)	Allelic	R	1.633(0.937-2.846)	0.08	89.8	≤0.01
			Caucasian		8	2710 (979/1731)	Dominant	R	1.371(0.882-2.131)	0.16	84.1	≤0.01
			Caucasian		5	1716 (588/1128)	Recessive	R	1.617(0.942-2.774)	0.08	59.8	0.04
GSTT1		null vs present	Caucasian	0.23	12	4279 (1393/2886)	Allelic	R	1.133(0.803-1.598)	0.48	74.4	≤0.01
NAT C282T	rs10419839	C > T	Caucasian	0.42	3	930 (372/558)	Dominant	R	0.752(0.209-2.515)	0.61	94.6	≤0.01
P53	rs1042522	G > C	Overall	0.35	10	3465 (1035/2430)	Allelic	R	1.146(0.833-1.577)	0.40	87.1	≤0.01
			Overall		11	3644 (1098/2546)	Dominant	R	1.000(0.681-1.469)	1.00	82.5	≤0.01
			Overall		10	3465 (1035/2430)	Recessive	R	1.294(0.739-2.268)	0.37	82.9	≤0.01
RET IVS1-126	rs2565206	G > T	Caucasian	0.30	3	1148 (332/816)	Allelic	R	1.227(0.899-1.673)	0.20	55.6	0.11
RET L769L	rs1800861	T > G	Overall	0.29	14	6665 (2793/3872)	Allelic	R	0.885(0.739-1.060)	0.19	75.8	≤0.01
			Overall		8	5802 (2342/3460)	Dominant	R	0.975(0.784-1.213)	0.82	67.7	≤0.01
			Overall		8	5802 (2342/3460)	Recessive	F	0.961(0.799-1.156)	0.67	0.0	0.71
XRCC1	rs25487	G > A	Overall	0.30	15	10252 (4217/6035)	Allelic	R	0.961(0.865-1.067)	0.45	61.5	≤0.01
			Overall		15	10252 (4217/6035)	Dominant	R	0.951(0.837-1.181)	0.44	55.5	≤0.01
			Overall		15	10252 (4217/6035)	Recessive	F	0.957(0.844-1.085)	0.49	28.0	0.15
XRCC1	rs25489	G > A	Overall	0.14	12	7996 (3168/4828)	Allelic	R	0.982(0.767-1.255)	0.88	81.8	≤0.01
			Overall		13	9757 (4033/5724)	Dominant	R	0.974(0.806-1.179)	0.79	64.5	≤0.01
			Overall		12	7996 (3168/4828)	Recessive	R	1.010(0.520-1.961)	0.98	78.6	≤0.01
XRCC1	rs1799782	C > T	Overall	0.19	12	8515 (3670/4945)	Allelic	R	1.112(0.899-1.375)	0.33	83.9	≤0.01
			Overall		12	8515 (3670/4945)	Dominant	R	1.054(0.837-1.328)	0.65	78.3	≤0.01
			Overall		12	8515 (3670/4945)	Recessive	R	1.364(0.866-2.151)	0.18	75.1	≤0.01
XRCC2 Arg188His	rs3218536	G > A	Overall	0.11	3	1380 (556/824)	Allelic	F	0.990(0.775-1.264)	0.94	3.3	0.36
XRCC3 A17893G	rs1799796	A > G	Overall	0.30	4	2477 (1106/1371)	Allelic	R	0.979(0.805-1.190)	0.83	58.4	0.07
			Overall		4	2477 (1106/1371)	Dominant	R	0.939(0.713-1.236)	0.65	64.1	0.04

Supplementary table 2 Insignificant variants in subtype analysis from meta-analysis

Gene	Variant	Alleles	Ethnicity	MAF[†]	studies	Number Evaluation sample size (case/control)	Genetic Models	effect model	Risk of Meta-Analysis OR(95%CI)	P_{value}	I(%)	P_Q
miR-146a	rs2910164	G > C	Caucasian	0.23	9	11653(2527/9126)	Allelic	F	1.058(0.979-1.145)	0.16	48.6	0.05
			Caucasian		9	11653(2527/9126)	Dominant	R	1.178(0.975-1.423)	≤0.01	66.0	≤0.01
			Caucasian		9	11653(2527/9126)	Recessive	R	0.745(0.487-1.140)	0.18	61.4	≤0.01
			Asian	0.49	3	5084(2330/2754)	Allelic	F	1.052(0.970-1.141)	0.22	0.0	0.53
			Asian		3	5084(2330/2754)	Dominant	R	1.071(0.935-1.226)	0.32	0.0	0.52
			Asian		3	5084(2330/2754)	Recessive	R	1.065(0.938-1.208)	0.33	0.0	0.75
P53	rs1042522	G > C	Caucasian	0.34	9	3125 (895/2230)	Allelic	R	1.065(0.783-1.450)	0.69	84.0	≤0.01
			Caucasian		10	3304 (958/2346)	Dominant	R	0.919(0.626-1.350)	0.67	79.9	≤0.01
			Caucasian		9	3125 (895/2230)	Recessive	R	1.175(0.655-2.108)	0.59	80.3	≤0.01
XRCC1	rs25487	G > A	Caucasian	0.34	10	6865 (2814/4051)	Allelic	R	0.938(0.857-1.027)	0.17	26.7	0.20
			Caucasian		10	6865 (2814/4051)	Dominant	R	0.916(0.802-1.046)	0.19	36.8	0.11
			Caucasian		10	6865 (2814/4051)	Recessive	F	0.919(0.795-1.063)	0.26	0.0	0.68
			Asian	0.26	5	3387 (1403/1984)	Allelic	R	1.024(0.783-1.339)	0.86	81.1	≤0.01
			Asian		5	3387 (1403/1984)	Dominant	R	1.033(0.778-1.373)	0.82	73.3	≤0.01
			Asian		5	3387 (1403/1984)	Recessive	F	1.078(0.841-1.383)	0.55	65.2	0.02
XRCC1	rs25489	G > A	Caucasian	0.09	8	4819 (1875/2944)	Allelic	R	0.999(0.657-1.517)	1.00	84.9	≤0.01
			Caucasian		9	6580 (2740/3840)	Dominant	R	0.991(0.748-1.314)	0.95	68.6	≤0.01
			Caucasian		8	4819 (1875/2944)	Recessive	R	1.012(0.281-3.642)	0.99	76.7	≤0.01
			Asian	0.20	4	3177 (1293/1884)	Allelic	R	1.003(0.816-1.233)	0.98	58.9	0.06
			Asian		4	3177 (1293/1884)	Dominant	R	0.970(0.756-1.245)	0.81	60.5	0.06
			Asian		4	3177 (1293/1884)	Recessive	R	1.256(0.927-1.700)	0.14	0.0	0.94
RCC3 A17893G	rs1799794	A > G	Asian	0.33	3	1621 (650/971)	Allelic	R	1.157(0.962-1.390)	0.12	34.2	0.22
			Asian		3	1621 (650/971)	Dominant	R	1.206(0.898-1.619)	0.21	50.4	0.13
			Asian		3	1621 (650/971)	Recessive	F	1.160(0.867-1.552)	0.32	0.0	0.84
RCC3 A17893G	rs1799796	A > G	Asian	0.29	3	1621 (650/971)	Allelic	R	1.073(0.916-1.257)	0.03	4.9	0.35
			Asian		3	1621 (650/971)	Dominant	R	1.079(0.833-1.319)	≤0.01	0.0	0.51
			Asian		3	1621 (650/971)	Recessive	R	1.079(0.833-1.319)	≤0.01	0.0	0.51
RET L769L	rs1800861	T > G	Caucasian	0.27	12	6224 (2602/3642)	Allelic	R	0.940(0.781-1.131)	0.51	74.3	≤0.01
RET S904S	rs1800863	C > G	Caucasian	0.19	9	3599 (1409/2190)	Allelic	R	1.054(0.864-1.286)	0.60	54.6	0.02
			Caucasian		5	2753 (1038/1715)	Dominant	R	1.132(0.842-1.521)	0.41	61.0	0.04
			Caucasian		5	2753 (1038/1715)	Recessive	F	1.442(0.958-2.171)	0.08	0.0	0.79

[illegible]
